# Probing Relevant Molecules in Modulating the Neurite Outgrowth of Hippocampal Neurons on Substrates of Different Stiffness

**DOI:** 10.1371/journal.pone.0083394

**Published:** 2013-12-30

**Authors:** Wei-Hsin Chen, Sin-Jhong Cheng, Jason T. C. Tzen, Chao-Min Cheng, Yi-Wen Lin

**Affiliations:** 1 Graduate Institute of Biotechnology, National Chung Hsing University, Taichung, Taiwan; 2 Department of Life Science and Institute of Zoology, National Taiwan University, Taipei, Taiwan; 3 Institute of Nanoengineering and Microsystems, National Tsing Hua University, Hsinchu, Taiwan; 4 Graduate Institute of Acupuncture Science, China Medical University, Taichung, Taiwan; 5 Acupuncture Research Center, China Medical University, Taichung, Taiwan; University of S. Florida College of Medicine, United States of America

## Abstract

Hippocampal neurons play a critical role in learning and memory; however, the effects of environmental mechanical forces on neurite extension and associated underlying mechanisms are largely unexplored, possibly due to difficulties in maintaining central nervous system neurons. Neuron adhesion, neurite length, and mechanotransduction are mainly influenced by the extracellular matrix (ECM), which is often associated with structural scaffolding. In this study, we investigated the relationship between substrate stiffness and hippocampal neurite outgrowth by controlling the ratios of polydimethylsiloxane (PDMS) base to curing agent to create substrates of varying stiffness. Immunostaining results demonstrated that hippocampal neurons have longer neurite elongation in 35∶1 PDMS substrate compared those grown on 15∶1 PDMS, indicating that soft substrates provide a more optimal stiffness for hippocampal neurons. Additionally, we discovered that pPKCα expression was higher in the 15∶1 and 35∶1 PDMS groups than in the poly-l-lysine-coated glass group. However, when we used a fibronectin (FN) coating, we found that pFAKy397 and pFAKy925 expression were higher in glass group than in the 15∶1 or 35∶1 PDMS groups, but pPKCα and pERK1/2 expression were higher in the 35∶1 PDMS group than in the glass group. These results support the hypothesis that environmental stiffness influences hippocampal neurite outgrowth and underlying signaling pathways.

## Introduction

Ramón y Cajal first investigated neuronal morphology, including neurite length, dendrite morphology, and characterization of the dendritic arbor more than 100 years ago [1]. These discoveries greatly increased the understanding of overall nervous system appearance and function. Neurite outgrowth and branching are highly complex processes that determine where nerve terminals will contact each other [1]–[5]. Hippocampal neuron outgrowth goes through five stages with significant distinguishable landmarks, from seeding to completion [6]–[8]. The first day after plating, lamellipodia form and adhere to the substrate and minor neurite formation is observed [9]. After 3 days of culturing, neuronal axons appear, followed by neurite branching at day 5. As the maturation process of hippocampal neurons continues through 7 days of culturing, neurite spines and higher order branches are formed [6]–[9].

Peripheral neurons can sense and respond to different external cues, such as mechanical stretching, compression, vibration, and touch [10]. Peripheral nerve terminals can convert mechanical input into transductive electrical signals for further responses [10]. During neurite initiation, focal adhesion complexes are formed, and microtubules align to develop a tense bundle, which is accompanied by the emergence of actin filaments to initiate a growth cone [11], [12]. Extracellular matrix (ECM) components including collagen, laminin, and fibronectin, act on surface membrane receptors to increase cell adhesion and neurite outgrowth [13], [14]. This signal further acts on focal adhesion kinase to ERK1/2 pathways to trigger actin filament transformation and microtubule neurite outgrowth [15], [16]. The integrin influences focal-adhesion formation and cell migration [17], [18], and also binds phosphatidylinositol 4, 5-bisphosphate (PIP2), in order to regulate protein kinase Cα (PKCα) activity [19]. PKC activation has been proved to be need for focal-adhesion formation and cell spreading in the integrin-mediated signaling cascade [20], [21], and regulated by Rho-family GTPase and focal adhesion kinase (FAK) [22]. Such mechanisms have been clarified in non-neuronal cell cultures, including those of 3T3 fibroblasts, epithelial cells, and cancer cells [15], [16]; Recent studies suggest that neurite outgrowth begins immediately after neuronal adhesion [23], [24]. Neurite extension is enhanced by the activation of membrane mechanical receptors and ECM [24]–[26]. The mechanic receptors on the membrane surface further induce intracellular focal adhesion kinase to alter microtubule and initiate neurite extension. Furthermore, PKC, FAK, and ERK signaling pathways further activate gene transcription to stabilize neurite formation [27], [28].

Scientists have developed materials and chemical scientific methods to provide an elastic context capable of mimicking the physiological conditions of living organisms [29], [30]. Examining results of cultured embryonic stem cells on PDMS gel of varying elasticity showed that environmental stiffness can alter cellular behaviors [31]. Among synthetic polymeric materials, hydrogels composed of polyacrylamide (PA) have been used to verify the effects of substrate flexibility on hippocampal neurite branching [32]. Flanagan et al. reported that spinal cord neurons grown on a softer substrate, sans coating with extracellular matrix molecules, formed more than three times as many branches as neurons grown on stiffer gels [33].

Polydimethylsiloxane (PDMS) is a ligand-coated synthetic polymeric system that can be used to alter stiffness to make materials of physiologically equivalent Young’s moduli [34]. PDMS has the advantage of being highly suitable for neuron growth because of its considerable flexibility and relatively low modulus. Chou et al. provided evidence that neuronal cells exhibit different neurite outgrowth on different substrates of varying stiffness [35]. Accordingly, PDMS is also useful for determine stretch-activated action potential in DRG neurons grown on PDMS substrate that is stretched via mechanical stimulation [36]. When DRG neurons are cultured on PDMS substrate coated with poly-l-lysine, neurons show lower cell density and neurite outgrowth [37].

Here, we studied the effects of elasticity on hippocampal neurite development. We hypothesized that our engineered system could alter substrate stiffness by varying ratio of base to curing agent, allowing us determine the preferred elasticity for hippocampal neurite formation. Our results indicate that hippocampal neurons have longer neurite growth on glass coverslips than on soft PDMS substrate. Within our PDMS substrate groups, hippocampal neurites were longest when cultured on 35∶1 PDMS substrate, indicating that this particular PDMS substrate offers a highly suitable environment for hippocampal neurite formation and extension. Our findings demonstrate that neurite lengths were correlated with pPKCα, pFAKy397, pFAKy925, and pERK1/2.

## Materials and Methods

### Polydimethlysiloxane

Polydimethlysiloxane (PDMS, Sylgard 184) was purchased from Dow Corning (Taipei, Taiwan) and the ratios of base to curing agent were modulated to approximate physiologically relevant Young’s modulus. We altered the ratios of PDMS base to curing agent (15∶1 35∶1, and 50∶1) to establish elastic moduli of approximately 173, 88, and 17 kPa [37], [38]. We used this capacity to produce various PDMS substrates and to determine which had elasticity that was more physiologically relevant than conventional glass (∼ 70 GPa).

### Hippocampal Primary Culture

**Figure 1:**
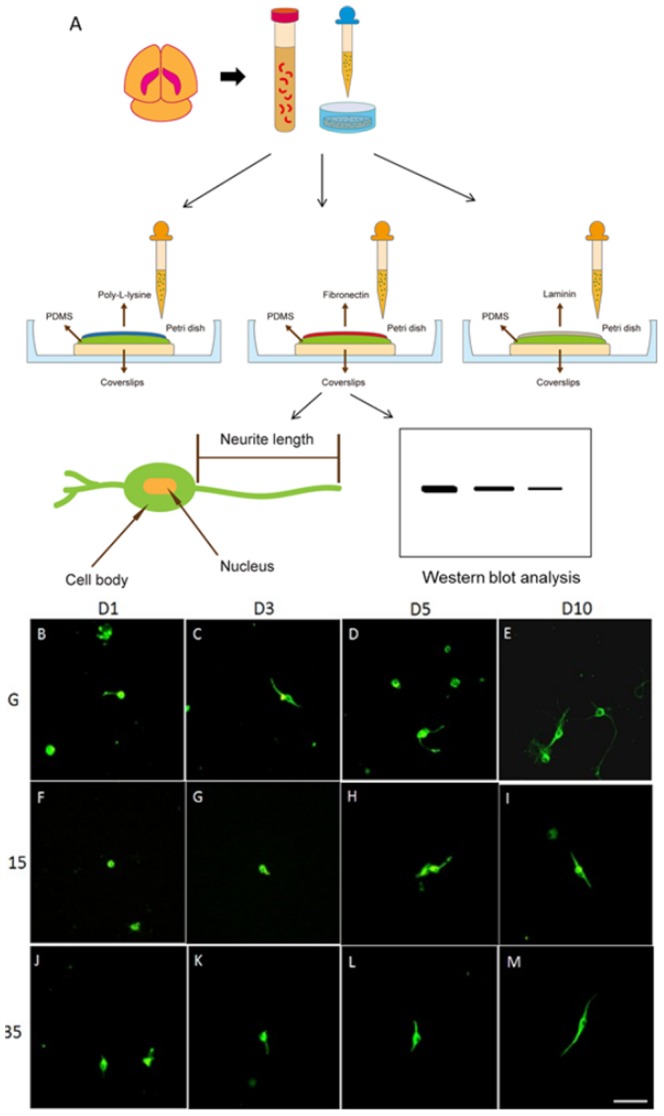
Schema of the experiment procedure and the Hippocampal primary cultures on glass, 15∶1 PDMS, and 35∶1 PDMS coated with poly-l-lysine, fibronectin or laminin over time followed staining with antibody or analyzing with western blot (A). Hippocampal neurons had short neurites 1 to 3 days after seeding on poly- l-lysine-coated glass coverslips (B, C). Hippocampal neurons had longer neurites 5 and 10 days after being seeded on poly- l-lysine-coated glass coverslips (D, E). The neurites were shorter 1 and 3 days after seeding on 15∶1 PDMS (F, G). Hippocampal neurons had longer neurites 5 and 10 days after seeding on 15∶1 PDMS (H, I). Hippocampal neurites at day 1 and day 3 after seeding on 35∶1 PDMS (J, K). Hippocampal neurons had longer neurites at 5 and 10 days after seeding on 35∶1 PDMS (L, M). Tubulin (green) was used to determine neurite lengths (scale bar  =  50 µm, n = 3).

Postnatal day 0 outbred ICR/CD1 mice (BioLASCO Taiwan Co., Ltd.) mice were used to obtain the hippocampal cultures. The usage of these animals was approved by the Institute of Animal Care and Use Committee of China Medical University, Taiwan, following the Guide for the Use of Laboratory Animals (National Academy Press). Mice were decapitated and brains were quickly removed. The bilateral hippocampi were dissected and placed in Neurobasal-A medium (Invitrogen, Carlsbad, CA, USA) at 4°C. The hippocampi were further transferred to 1.4 mL papain solution (∼100 units/mL papain, 0.5 mM EDTA, 0.2 mg/mL cysteine; Sigma, St. Louis, MO, USA) 37°C for 15 min for digestion. Following this, we added 0.263 mL DNasI (0.21%; Sigma, St. Louis, MO, USA) and 0.182 mL of MgCl_2_ (12.5 mM; Sigma, St. Louis, MO, USA) to the solution incubated at 37°C 5 min, then added 0.185 mL of horse serum. The cells were pelleted by centrifugation at 1500 rpm for 5 min. 4 mL of Neurobasal-A medium was added to the cell pellets. This solution/suspension was triturated with a 10 mL pipette approximately 50 times then passed through a 100 µm filter. The hippocampal neurons were then seeded on either glass coverslips or PDMS coated with poly-L-lysine (0.1%; Sigma, St. Louis, MO, USA), fibronectin (10 μg/mL; Sigma, St. Louis, MO, USA) or laminin (1 μg/mL; Millipore, Billerica, MA, USA) at a density of 1×10^5^ cells/mL. Coverslips with hippocampal neurons were then placed in petri dishes with Neurobasal-A medium containing B-27 supplement (Invitrogen, Carlsbad, CA, USA) and 1% penicillin/streptomycin. The hippocampal neurons were incubated at 5% CO_2_ at 37°C for 1-day, 3-day, 5-day, and 10-day time spans for further experiments. All procedures were shown as Figure 1A.

### Immunofluorescent Microscopy and Image Analysis

After culturing hippocampal neurons on different substrates for different time spans, hippocampal neurons were washed with phosphate buffer saline (PBS) three times, fixed with 4% paraformaldehyde, and incubated with PBS containing 1% bovine serum albumin, 0.1% Triton X-100, and 0.02% sodium azide for blocking. The samples were further mixed with a primary antibody at 4°C overnight. The primary antibody used was anti-beta Tubulin (1∶500, Novus Biologicals, Littleton, CO, USA). The secondary antibody was incubated with Goat anti-Rabbit IgG DyLight488 (1∶1000, Jackson ImmunoResearch Laboratories, West Grove, PA). The stained hippocampal neurons were mounted with glycerol, sealed under a coverslip, and then examined using fluorescent microscope (Olympus, BX-51, Japan). The images were further analyzed using NIH ImageJ software (Bethesda, MD USA). Six primary cultures in total were employed, and twenty images were captured for each group. Neurite length was calculated from cell body to nerve terminal end as shown in Figure 1A.. Neurite outgrowth percentage was calculated by dividing the neurons with neurites by total neurons.

### Western blot analysis

Cultured hippocampal neuron proteins were extracted on days 1, 3 and 5. Total proteins were prepared by homogenizing neurons in lysis buffer containing 50 mM Tris-HCl pH 7.4, 250 mM NaCl, 1% NP-40, 5 mM EDTA, 50 mM NaF, 1 mM Na3VO4, 0.02 % NaN3 and 1× protease inhibitor cocktail (Amresco, Solon, USA). The extracted proteins (30 µg per sample assessed by BCA protein assay) were subjected to 8% SDS-Tris glycine gel electrophoresis and transferred to a PVDF membrane. The membrane was blocked with 5% nonfat milk in TBS-T buffer (10 mM Tris pH 7.5, 100 mM NaCl, 0.1% Tween 20), incubated with anti-PKCα [pSer657] (1∶1000, Millipore, Billerica, MA, USA), pFAK [pTyr397] (1∶500, Novus Biologicals, Littleton, CO, USA), pFAK [pTyr925] (1∶500, Novus Biologicals, Littleton, CO, USA), and pERK1/2 [pThr202, pTyr204] (1∶500, Novus Biologicals, Littleton, CO, USA) in TBS-T with 1% bovine serum albumin, and incubated for 1 hour at room temperature. Peroxidase-conjugated anti-rabbit antibody and anti-mouse antibody (1∶5000, Jackson ImmunoResearch Laboratories, West Grove, PA) were used as a secondary antibody. The bands were visualized by an enhanced chemiluminescencent substrate kit (Thermo Scientific, Waltham, MA, USA) with LAS-3000 Fujifilm (Fuji Photo Film Co. Ltd). Where applicable, the image intensities of specific bands were quantified with NIH ImageJ software (Bethesda, MD, USA).

### Statistical analysis

All statistic data are presented as the mean ± standard error. Statistical significance between glass and PDMS group was tested using the ANOVA test, followed by a post hoc Tukey’s test (*p*<0.05 was considered statistically significant).

## Results

The hippocampus is known to play significant roles in memory processing but has rarely been associated with mechanical stimulation. Our recent studies reported that 35∶1 soft PDMS substrate could be used to mimic physiological conditions and examine mechanical responses among and within DRG neurons [36], [37]. Because external substrate elasticity imposed on DRG neurons can alter the responses of cytoskeletal elements, we were motivated to examine hippocampal neurite outgrowth. To identify the effects of substrate elasticity on hippocampal neurite outgrowth, we first verified neurite length by culturing hippocampal neurons on glass coverslips coated with poly-l-lysine (staining with microtubules for cytoskeleton). Poly-l-lysine is known to increase neuron attachment while accelerating cell growth [39]. In our glass coverslip group, hippocampal neurite appearance and total length were relatively short 1 day after seeding (Fig. 1B), yet after 3 days, the neurite length was longer (Fig. 1C). Neurite lengths increased from day 3 to day 5, and continued to lengthen even after 10 days of culturing on glass coverslips (Fig. 1D, E). When hippocampal neurons were cultured on 15∶1 PDMS substrate, neurites were short from day 1 to day 3 (Fig. 1F, G). Similar to the phenomena observed in the 15∶1 PDMS group, neurites were longer from day 5-10 but still significantly shorter compared with grown on stiff glass (Fig. 1H, I). Next, we examined neurite length of hippocampal neurons cultured on softer PDMS substrate. Our results showed that neurites were observed on days 1 and 3 after plating on softer 35∶1 PDMS (Fig. 1J, K), and neurite outgrowth increased 5 or 10 days after seeding (Fig. 1L, M).

**Figure 2:**
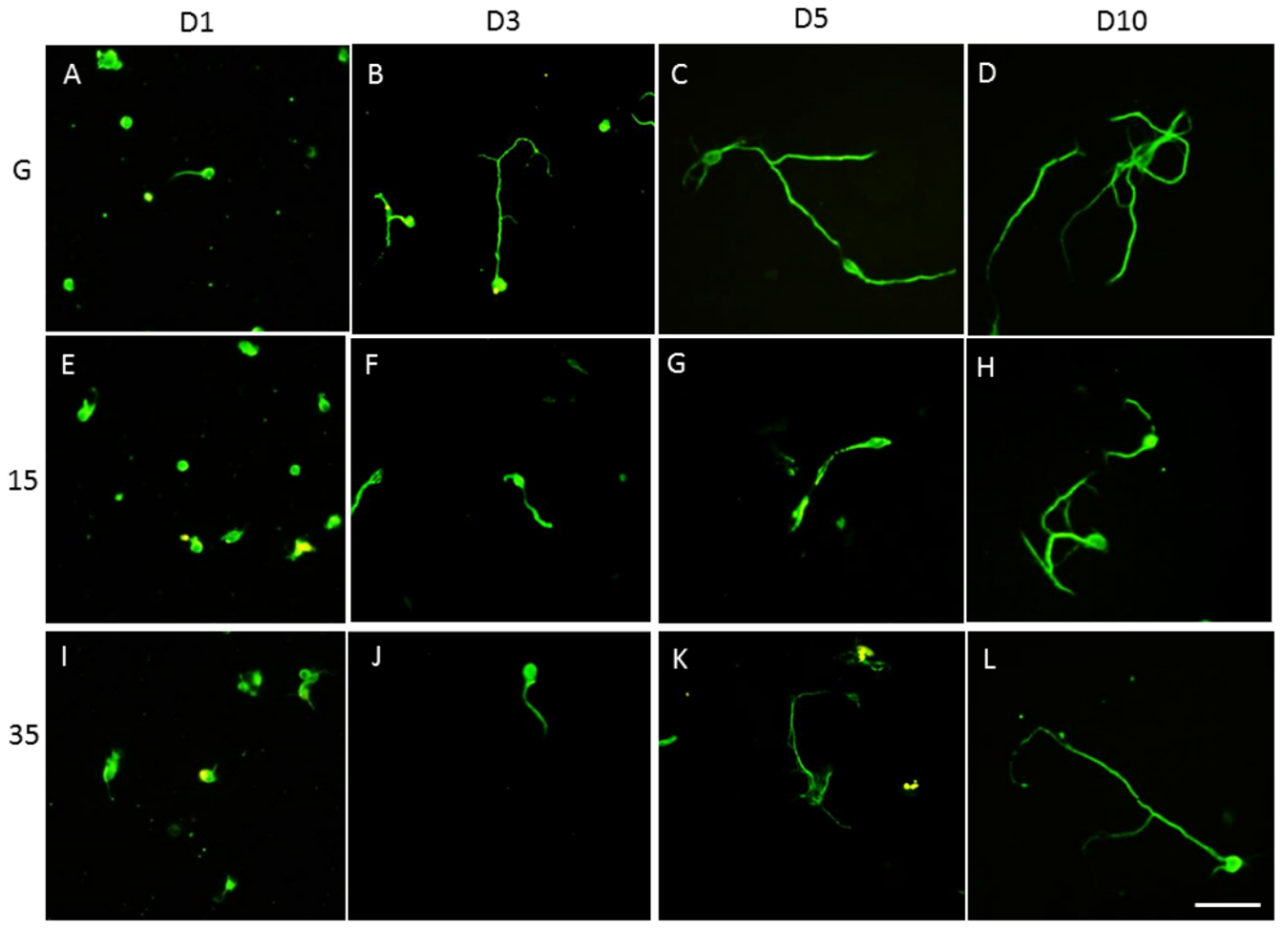
Hippocampal primary cultures on glass, 15∶1 PDMS, and 35∶1 PDMS coated with fibronectin over time. Hippocampal neurons had short neurites 1 and 3 days after seeding on fibronectin-coated glass (A, B). Hippocampal neurites were longer 5 and 10 days after seeding on glass coverslips coated with fibronectin (C, D). The neurites were shorter 1 and 3 days after seeding on 15∶1 PDMS (E, F). Hippocampal neurons had longer neurites at 5 and 10 days after seeding on 15∶1 PDMS (G, H). Hippocampal neurites at 1 and 3 days after seeding on 35∶1 PDMS (I, J). Hippocampal neurons had longer neurites at 5 and 10 days after seeding on 35∶1 PDMS (K, L). Tubulin (green) was used to determine neurite lengths (scale bar  =  50 µm, n = 3).

ECM (including FN) is well known for its ability to enhance cell adhesion and support neurite outgrowth and axonal regeneration [40]. We sought to identify the effects of FN on hippocampal neurite outgrowth, particularly when cultured on soft PDMS substrate. Hippocampal neurite growth was initiated 1 day after seeding in our glass coverslip group, and outgrowth was quickly established by day 3 (Fig. 2A, B). Neurite lengths and branches increased significantly with time (5 to 10 days for neurons plated on glass coverslips) (Fig. 2C, D). When culturing hippocampal neurons on 15∶1 PDMS, neurites also developed from days 1–3 (Fig. 2E, F) to days 5–10 (Fig. 2G, H). Similarly, neurite formation proceeded properly with short neurite formation on days 1 and 3 after culturing neurons on 35∶1 PDMS (Fig. 2I, J). Hippocampal neurite formation increased with longer incubation, i.e., from 5 to 10 days (Fig. 2K, L).

**Figure 3:**
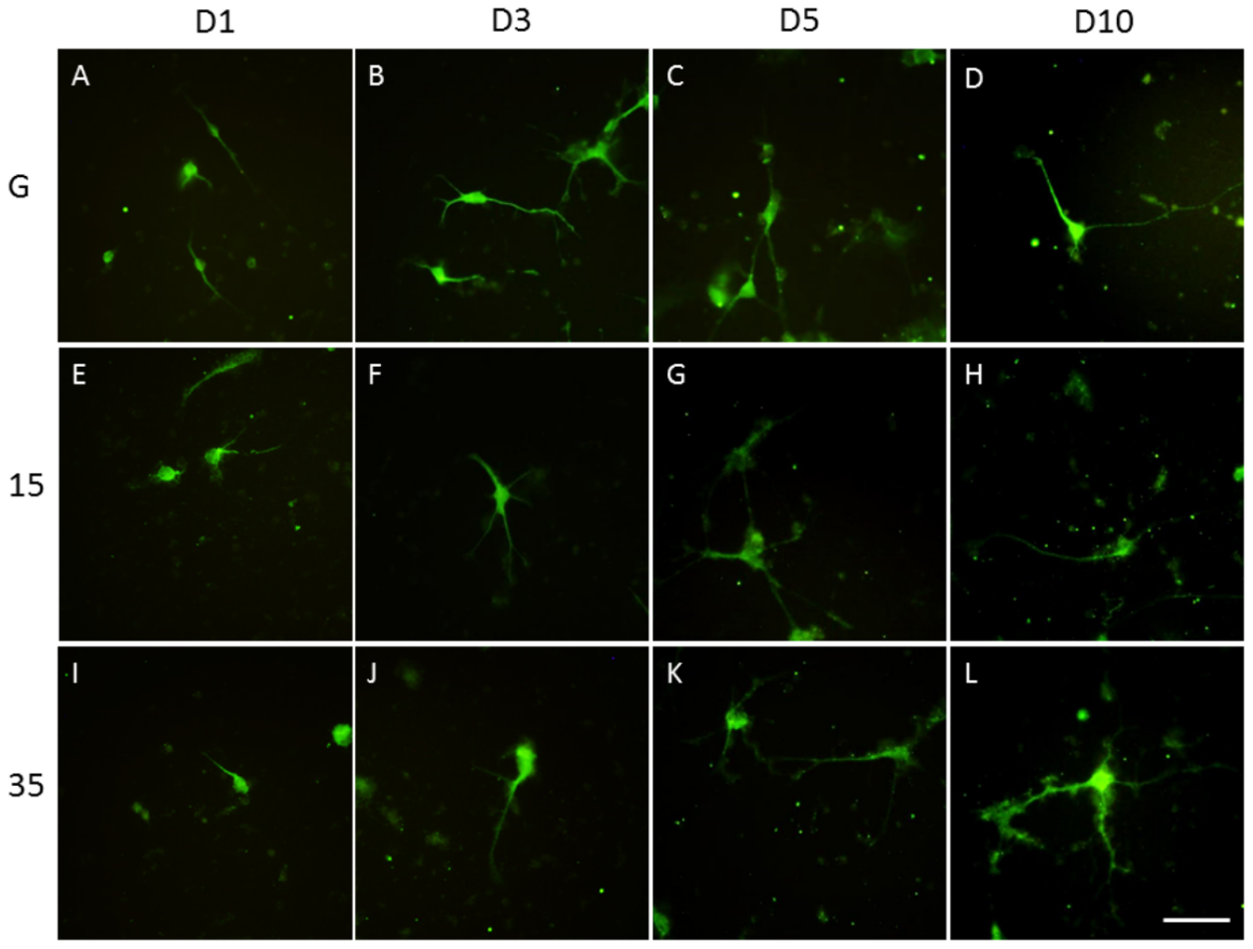
Hippocampal primary cultures on glass, 15∶1 PDMS, and 35∶1 PDMS coated with laminin over time. Hippocampal neurons had short neurites 1 and 3 days after seeding on laminin-coated glass (A, B). Hippocampal neurites were longer 5 and 10 days after seeding on glass coverslips coated with laminin (C, D). The neurites were shorter 1 and 3 days after seeding on 15∶1 PDMS (E, F). Hippocampal neurons had longer neurites at 5 and 10 days after seeding on 15∶1 PDMS (G, H). Hippocampal neurites at 1 and 3 days after seeding on 35∶1 PDMS (I, J). Hippocampal neurons had longer neurites at 5 and 10 days after seeding on 35∶1 PDMS (K, L). Tubulin (green) was used to determine neurite lengths (scale bar  =  50 µm, n = 3).

Laminin is an ECM widely used for cell attachment and crucial for neuronal survival [41]. We cultured the hippocampal neurons on laminin to determine neurite outgrowth on different subtracts. Hippocampal neurite growth was initiated 1 day after seeding in our glass coverslip group, and outgrowth was quickly established by day 3 (Fig. 3A, B). Neurite lengths and branches increased significantly with time (5 to 10 days) for neurons plated on glass coverslips (Fig. 3C, D). When culturing hippocampal neurons on 15∶1 PDMS, neurites also developed from days 1–3 (Fig. 3E, F) to days 5–10 (Fig. 3G, H). Similarly, neurite formation proceeded properly with short neurite formation on days 1 and 3 after culturing neurons on 35∶1 PDMS (Fig. 3I, J). Hippocampal neurite formation increased with longer incubation, i.e., from 5 to 10 days (Fig. 3K, L).

**Figure 4:**
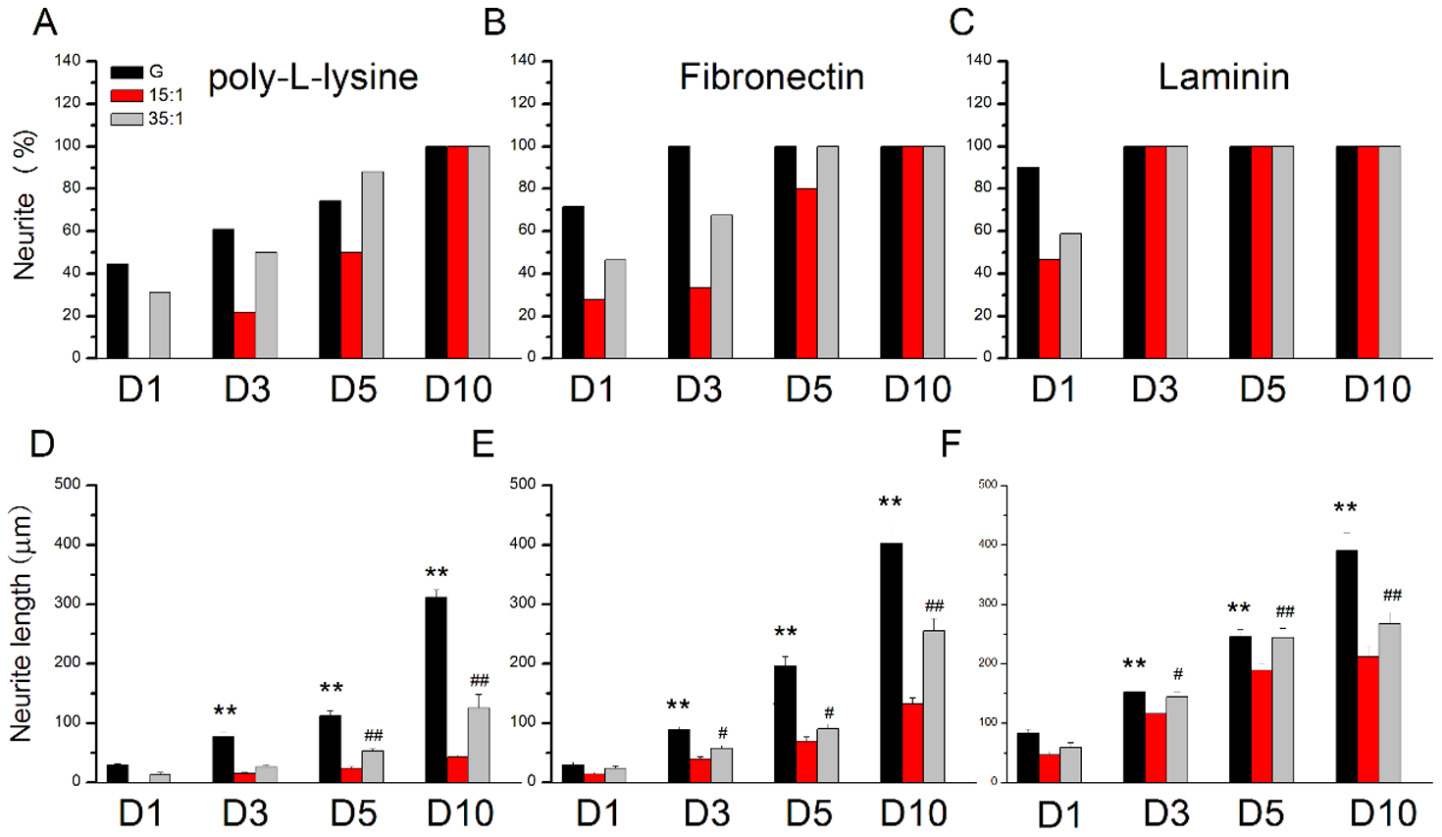
Quantification of the percentage of neurite outgrowth and neurite length on substrates with differing elasticity over time. (A) Hippocampal neurons with neurite were determined for cultures on glass substrates, 15∶1 PDMS and 35∶1 PDMS substrates coated with poly- l-lysine. (B) Percentage of neurite outgrowth over time on substrates with varying elasticity, coated with fibronectin. (C) Percentage of neurite outgrowth over time on substrates with varying elasticity, coated with laminin. (D) Hippocampal neurons appear to have relatively short neurites when grown on 15∶1 PDMS substrates coated with poly- l-lysine. (E) The neurite lengths of hippocampal neurons were determined while culturing hippocampal neurons on glass substrates, 15∶1 and 35∶1 PDMS coated with fibronectin. Hippocampal neurite length increased when seeding on glass substrate, 15∶1and 35∶1 PDMS coated with fibronectin. (F) Hippocampal neurons appear to have relatively short neurites when grown on 15∶1 PDMS substrates coated with laminin. (*p<0.05 compared with glass substrate, **p<0.01 compared with a glass substrate, ^#^ *p*<0.05 compared with 35∶1 PDMS, ^##^ *p*<0.01 compared with 35∶1 PDMS, n = 50 per groups). The error bars represent s.e.m.

The results were quantified and plotted (Fig. 4A–F). Over time, the percentages of neurite initiation increased for cultures initiated on glass coverslips, from 1 to 10 days (Fig. 4A). Neurite length also increased over time, from 1 to 10 days, after culturing on solid glass substrate coated with poly-l-lysine. Similar phenomena were obtained for cultures in the soft PDMS groups (from 15∶1 to 35∶1 PDMS) (Fig. 4D). To determine the effects of ECM on hippocampal neurite performance, we coated FN to enhance focal adhesion. Our results show that the percentage of neurite formation was augmented when FN was used in all substrate groups from day 1 to day5 (Fig. 4B). Furthermore, hippocampal neurites showed a relative increase in length (after plating on FN-coated substrates) compared with the poly-l-lysine group (Fig. 4E). We also calculated the neurite initiation percentage of the cells cultured on coated with laminin (Fig. 4C). Furthermore, hippocampal neurites showed a relative increase in length (after plating on laminin-coated substrates) compared with the poly-l-lysine group, especially in the PDMS groups (Fig. 4F).

**Figure 5:**
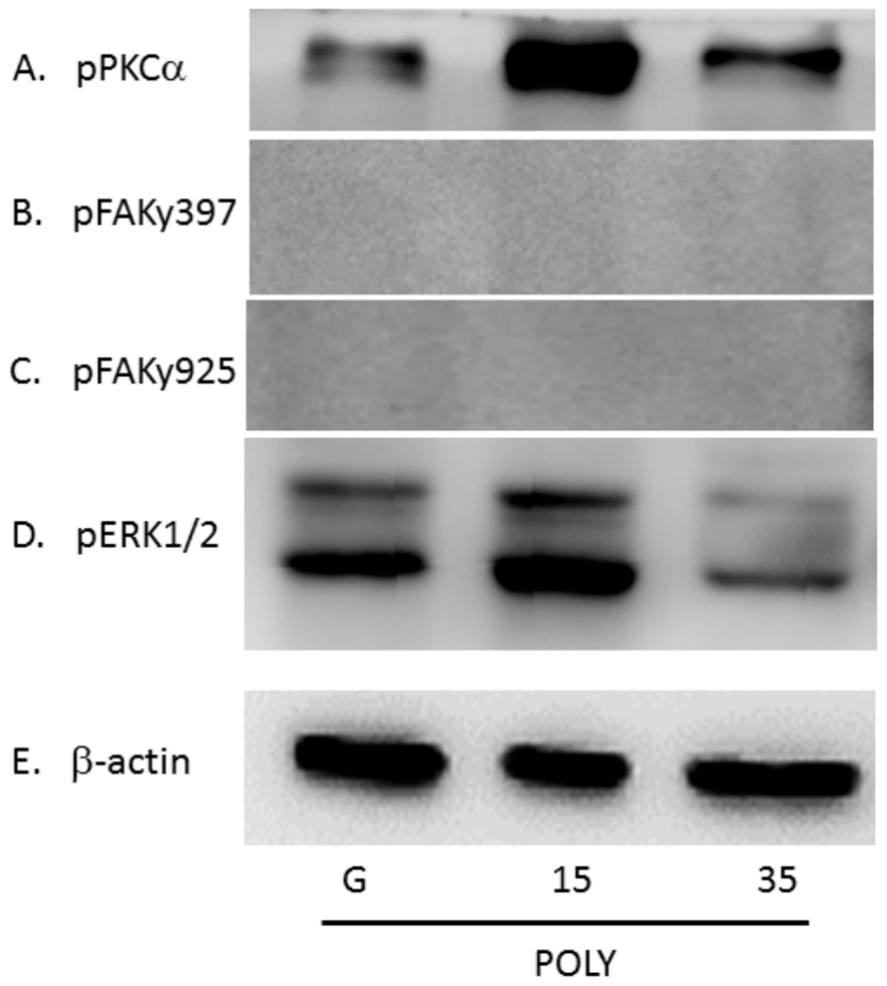
pPKCα, pFAKy397, pFAKy925, and pERK1/2 were quantified 5 days after plating on substrates coated with poly-l-lysine. After culturing for 5 days, hippocampal neuron lysates were reacted with (A) pPKCα, (B) pFAKy397, (C) pFAKy925, (D) pERK1/2 antibodies, and (E) β-actin. Three groups shown: glass, 15∶1 PDMS, and 35∶1 PDMS. (n = 3)

Understanding and accounting for the response of hippocampal neurite formation for cultures grown on softer substrates is critical in understanding the effects of mechanical environmental stimulation. Neurite length and extension proliferation is highly associated with mechanical force-activated kinases, such as pPKCα, pFAK397, pFAK925, and pERK1/2 [42]. We examined whether substrate stiffness altered focal adhesion and neurite elongation. We cultured hippocampal neurons on either glass coverslips or soft (15∶1 and 35∶1) PDMS coated with poly-l-lysine, a non-selective focal adhesion activator, to determine kinase expression. While culturing hippocampal neurons on substrates with varying stiffness, the expression of pPKCα was relatively increased with short neurite formation in the 15∶1 PDMS group (Fig. 5A; 131.46%±9.01% compared with glass groups, n = 3, P<0.05) and 35∶1 PDMS group (Fig. 5A; 127.74%±18.16% compared with glass groups, n = 3, P<0.05). We further discovered that pFAK397 and pFAK925 levels were not detectable in all groups (Fig. 5B, C). Our results also indicated that pERK1/2 was highest in the 15∶1 soft PDMS group (Fig. 5D; 123.59%±23.27% compared with glass groups, n = 3, P<0.05) and in 35∶1 PDMS group (Fig.5D; 105.48%±11.39% compared with glass groups, n = 3, P > 0.05). Similar results were observed in day 1 and day3. While culturing hippocampal neurons (day 1) on substrates coated with poly-L-lysine, the expression of pPKC α was relatively increased with short neurite formation in the 15∶1 PDMS group (Fig. S1; 196.88%±19.04% compared with glass groups, n = 3, P<0.05) and 35∶1 PDMS group (Fig. S1; 385.76%±13.87% compared with glass groups, n = 3, P<0.05). We further discovered that pFAK397 and pFAK925 levels were not detectable in all groups (Fig. S1). Our results also indicate that pERK1/2 was highest in the 15∶1 soft PDMS group coated with poly-L-lysine (Fig. S1; 164.89%±16.56% compared with glass groups, n = 3, P<0.05) and in 35∶1 PDMS group (Fig. S1; 186.86%±47.16% compared with glass groups, n = 3, P<0.05). While culturing hippocampal neurons (day 3) on substrates coated with poly-L-lysine, pPKC was also increased in both 15∶1 and 35∶1 groups (Fig. S2). All results were quantified and plotted as a bar chart in Figures S3 and S4. The group using glass coated with poly-L-lysine was used as a baseline.

**Figure 6:**
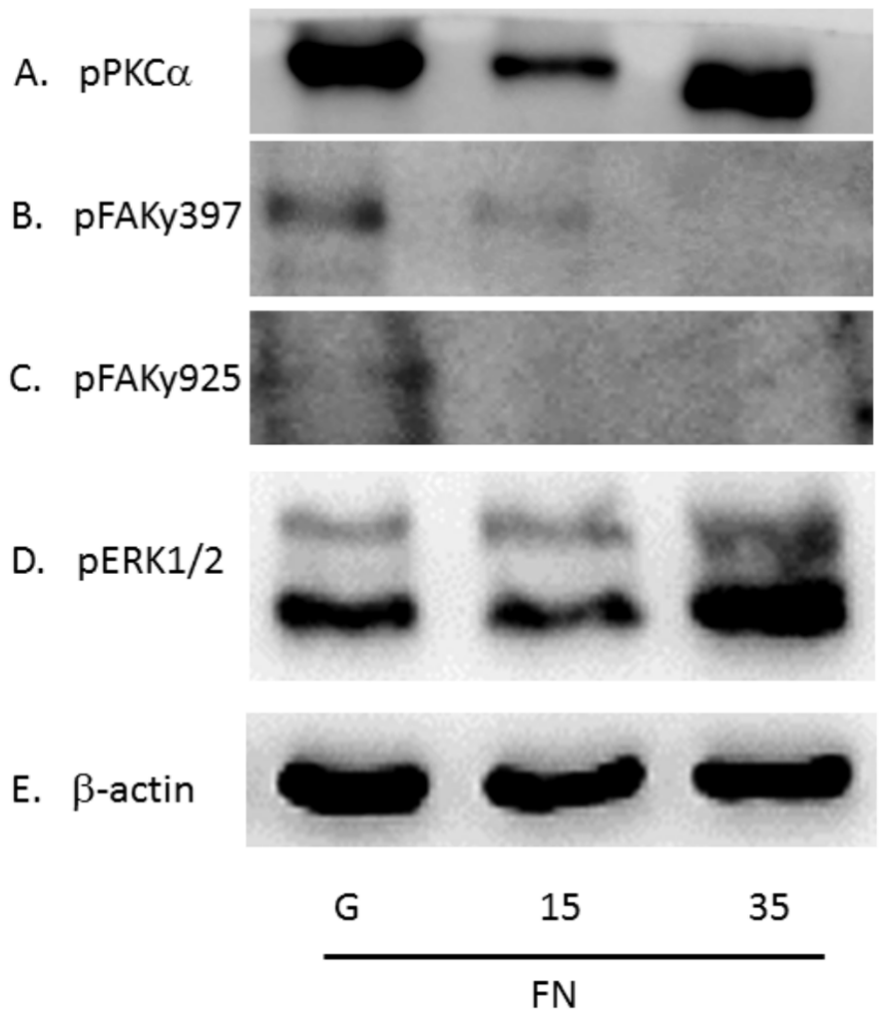
pPKCα, pFAKy397, pFAKy925, and pERK1/2 were quantified 5 days after plating on substrates coated with fibronectin. After culturing for 5 days, hippocampal neurons lysates were reacted with (A) pPKCα, (B) pFAKy397, (C) pFAKy925, (D) pERK1/2 antibodies, and (E) β-actin. Three groups shown: glass, 15∶1 PDMS, and 35∶1 PDMS. (n = 3)

**Figure 7:**
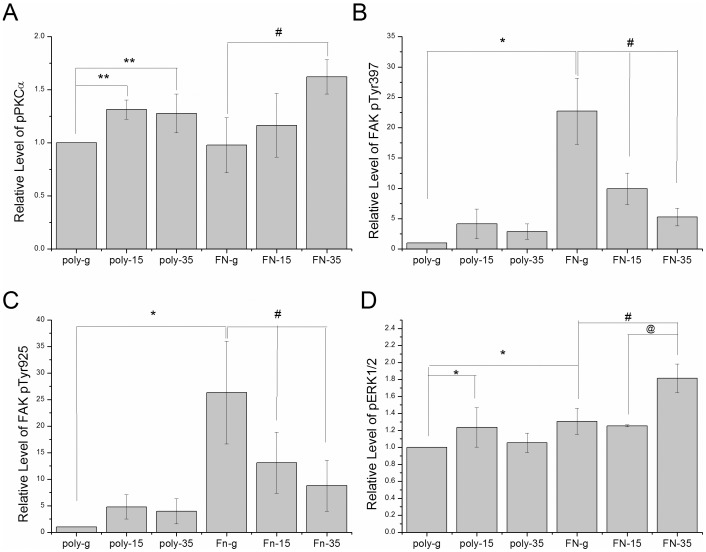
Quantification of Western blot results (A) pPKCα, (B) pFAKy397, (C) pFAKy925, (D) pERK1/2 antibodies, Six groups shown: glass, 15∶1 PDMS, and 35∶1 coated with poly-L-lysine or fibronectin. (*p<.05 compared to poly-g groups, # *p*< .05 compared to FN-g groups, @ *p*<.05 compared to FN-15 groups)

Furthermore, we examined the above-mentioned pathways by culturing hippocampal neurons on FN-coated substrates. FN activates the highest expression of pPKCα in neurons in the 35∶1 PDMS compared with the glass groups (Fig. 6A; 35∶1 as 162.16%±16.1% compared with glass groups, n = 3, P<0.05). It is noteworthy that pFAK397 and pFAK925 were increased in glass group after seeding on FN-coated substrate (Fig. 6B, C). The level of pERK1/2 was simultaneously augmented with the same FN manipulation and demonstrated a proliferative increase in our 35∶1 PDMS groups (Fig. 6D; 181.34%±16.9% compared with glass groups, n = 3, P<0.05). The results were quantified and plotted as a bar chart in Figure 7, the group using glass coated with poly-l-lysine was used as a baseline.

## Discussion

The current study demonstrates the effects of soft matrix elasticity on hippocampal neurite formation and extension, which is highly regulated by ECM. To accomplish this, we employed PDMS substrate to control the stiffness and force for hippocampal neurite elongation. This approach was used to examine the cytoskeletal microtubule protein response of hippocampal neurons. Our findings are important because they elucidate the response of hippocampal neurons grown on various substrates of varying elasticity and indicate that neurite elongation is greatest on glass coverslips, which was verified by microtubule staining. Interestingly, hippocampal neurons seeded on PDMS substrate demonstrated shorter neurite length. From our array of PDMS substrates (15∶1, 35∶1, and 50∶1), the most suitable elasticity for hippocampal neuron growth was 35∶1 PDMS soft substrate. Among our three ratios of PDMS, 50∶1 PDMS was not suitable for cell growth and induced a low survival rate. (Data not shown). We also determined that pPKCα expression was relatively high for cultures grown on 15∶1 or 35∶1 PDMS compared with that for cultures grown on glass surfaces coated with poly-l-lysine. We found that pFAKy397 and pFAKy925 were expressed at low levels or not at all in neurons seeded on poly-l-lysine-coated substrate. We also determined that FN activated the highest expression levels of pPKCα and pERK1/2 in 35∶1 PDMS groups compared with glass groups. It is noteworthy that FAKs were induced in cultures grown in our glass group after seeding on FN-coated substrate. We consider that these results are significant and relevant to investigating cell–material interactions, cell mechanics, and biological engineering.

Previously published work reported that substrate stiffness can affect hippocampal neurites by altering gel rigidities from 0.02%–0.6% bis-acrylamide, and neuronal density can be changed by culturing on substrates with varying stiffness [43]. The aforementioned published work also showed that cell density can influence neurite branching when neurons are seeded on soft or stiff gels. One report described that the number of primary and secondary dendrites and branch and terminal points were higher in neurons cultured on stiff gels [32]. Our results indicated that hippocampal neurons cultured on solid glass coverslips exhibit longer neurite extension compared with those cultured on soft PDMS. Within our PDMS substrate groups, hippocampal neurons developed longer neurites on 35∶1 PDMS compared with 15∶1 or 50∶1. This implies that neurons are able to adhere to and initiate neurite growth on suitably rigid environments. Findings showing hippocampus cultured on FN coating groups with longer neurite length compared with poly-l-lysine groups suggest that FN can activate membrane mechanical receptors, possibly through integrin to enhance the focal adhesion complex and further increase neurite elongation, which is accompanied by increased pFAK, and pERK1/2 phosphorylation. Our findings are crucial because they elucidate the response of hippocampal neurite length on soft and firm substrate matrices and detail molecular mechanisms that support underlying growth and elongation mechanisms.

Both central and peripheral neurons have the ability to sense external environmental stiffness. Our previous study indicated that DRG neurons tend to attach and initiate neurite growth when cultured on a particular soft PDMS [37]. Flanagan et al. reported that mechanical forces have significant effects on spinal cord neuronal morphology and neurite branching [33]. They also suggested that neurons can respond to substrate stiffness and alter neurite branching. While neurons have been successfully cultured on softer substrates, spinal cord neurons, in particular, formed more than thrice more quickly and on softer substrates compared with stiffer gels [33].

## Conclusions

It is well known that cells can respond to environmental stiffness and multiple molecular cues during neurite extension, but few studies have investigated the relevant signaling pathways. Here, we suggest that hippocampal neurons show improved attachment and neurite extension on substrates with specific stiffness (35∶1 PDMS). FN can induce hippocampal neurons to elongate neurite length, which is accompanied by increased pFAKs, and pERK1/2 phosphorylation compared to poly-L-lysine groups. These relative signaling pathways indicate crucial effects in hippocampal neuron regeneration and neurite development. This work is important in fields including neuroscience, biomaterials, and neuron–material interactions.

## Supporting Information

Figure S1 **pPKCα, pFAKy397, pFAKy925, and pERK1/2 were quantified 1 days after plating on substrates coated with poly-L-lysine or fibronectin**. After culturing 1 day, hippocampal neuron lysates were reacted with (A) pPKCα, (B) pFAK397, (C) pFAK925, (D) pERK1/2 antibodies, and (E) β-actin. Three groups shown: glass, 15:1 PDMS, and 35:1 PDMS. (n = 3).
(TIF)

Figure S2 **pPKCα, pFAKy397, pFAKy925, and pERK1/2 were quantified 3 days after plating on substrates coated with poly-L-lysine or fibronectin.** After culturing 3 days, hippocampal neuron lysates were reacted with (A) pPKCα, (B) pFAKy397, (C) pFAKy925, (D) pERK1/2 antibodies, and (E) β-actin. Three groups shown: glass, 15:1 PDMS, and 35:1 PDMS. (n = 3).
(TIF)

Figure S3 **Quantification of western blot results (A) pPKCα, (B) pERK1/2 antibodies, and six groups shown: glass, 15:1 PDMS, and 35:1 coated with poly-L-lysine or fibronectin.** (*P<.05 compared to poly-g groups, @@ P<.01 compared to FN-15 groups).
(TIF)

Figure S4 **Quantification of western blot results (A) pPKCα, (B) pERK1/2 antibodies, and six groups shown: glass, 15:1 PDMS, and 35:1 coated with poly-L-lysine or fibronectin.** (*P<.05 compared to poly-g groups, # P<.05 compared to FN-g groups).
(TIF)
