# Supplementary figures and images for: Probing Relevant Molecules in Modulating the Neurite Outgrowth of Hippocampal Neurons on Substrates of Different Stiffness
Source: PLoS One. 2013 Dec 30;8(12):e83394. doi: 10.1371/journal.pone.0083394 (PMC3875460; doi:10.1371/journal.pone.0083394)

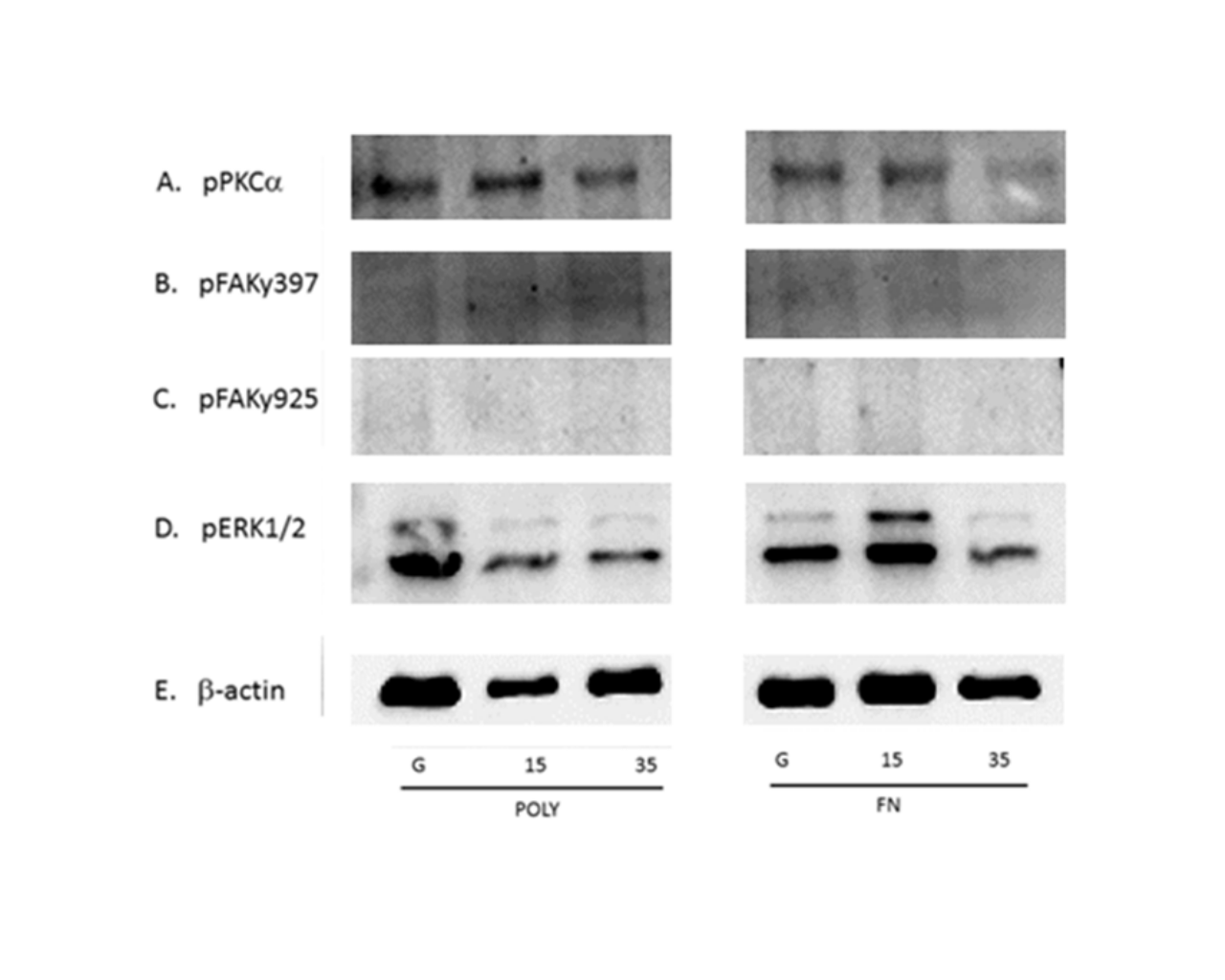

Supplement: Figure S2 — pPKCα, pFAKy397, pFAKy925, and pERK1/2 were quantified 3 days after plating on substrates coated with poly-L-lysine or fibronectin. After culturing 3 days, hippocampal neuron lysates were reacted with (A) pPKCα, (B) pFAKy397, (C) pFAKy925, (D) pERK1/2 antibodies, and (E) β-actin. Three groups shown: glass, 15:1 PDMS, and 35:1 PDMS. (n = 3). (TIF) [file pone.0083394.s002.tif]
